# Paeoniflorin Attenuates APAP-Induced Liver Injury via Intervening the Crosstalk Between Hepatocyte Pyroptosis and NETs

**DOI:** 10.3390/ijms26041493

**Published:** 2025-02-11

**Authors:** Yu-Ru Zhu, Ya-Qin Yang, Dan-Dan Ruan, Yue-Mei Que, Hang Gao, Yan-Zi Yang, Hua-Jun Zhao

**Affiliations:** 1 School of Pharmaceutical Sciences, Zhejiang Chinese Medical University, Hangzhou 311402, China; zyr@zcmu.edu.cn (Y.-R.Z.); rc.yaqin0902@zcmu.edu.cn (Y.-Q.Y.); 202321126811210@zcmu.edu.cn (D.-D.R.); 202321124011142@zcmu.edu.cn (Y.-M.Q.); 2 Academy of Chinese Medical Sciences, Zhejiang Chinese Medical University, Hangzhou 310053, China; 20211112@zcmu.edu.cn

**Keywords:** liver injury, acetaminophen, pyroptosis, neutrophil extracellular traps, paeoniflorin

## Abstract

(1) Liver injury caused by an overdose of acetaminophen (APAP) represents a major public health concern. Paeoniflorin (PF) has been reported to have anti-inflammatory and liver-protective effects, but the underlying mechanisms remain unclear. This study aimed to investigate the effect of PF on the crosstalk between pyroptosis and NETs in AILI. (2) APAP-treated *C57BL/6J* mice were used to demonstrate the protective effect of PF on liver injury. HepG2 and dHL-60 cells were cultured to study the effects of PF on hepatocyte pyroptosis and neutrophil extracellular traps (NETs) in vitro. Moreover, cell co-culture experiments were performed, and mice were treated with a NETs-depleting agent and hepatocyte pyroptosis inhibitor to investigate the improvement of AILI induced by PF through regulating the crosstalk between hepatocyte pyroptosis and NETs. (3) PF significantly alleviated AILI. Additionally, PF inhibited the expression of pyroptosis-related proteins, high-mobility group box 1 (HMGB1), and NETs-associated proteins in vitro and in vivo. The co-culture experiments demonstrated that PF not only inhibited the NETs triggered by hepatocyte pyroptosis, but also suppressed the hepatocyte pyroptosis induced by NETs. In mice with depleted neutrophils, the level of hepatocyte pyroptosis notably decreased, indicating a diminished impact of PF. Similarly, NETs formation was reduced in mice receiving a pyroptosis inhibitor compared to the APAP group. Compared with DNase I alone, the reduction effect of PF combined with DNase I on serum ALT and AST levels decreased from 46.857% and 39.927% to 44.347% and 33.419%, respectively. Compared with DSF alone, PF combined with DSF reduced the ALT and AST levels from 46.857% and 39.927% to 45.347% and 36.419%, respectively. (4) PF demonstrated therapeutic effects on AILI. Its mechanism involves the regulation of the crosstalk between hepatocyte pyroptosis and NETs. This research substantiates the pharmacological promise of PF as a therapeutic intervention for acute AILI.

## 1. Introduction

Drug-induced liver injury (DILI) has emerged as the leading cause of acute liver failure (ALF) [1]. Acetaminophen (APAP) is the most commonly used tool for studying drug-induced liver injury (DILI), as its use provides a well-characterized and reproducible system for investigating hepatotoxicity mechanisms. APAP-induced liver injury (AILI) is a leading cause of acute liver failure worldwide, making it a significant model for translational research and therapeutic development [2]. Although APAP is considered safe at therapeutic doses, excessive or prolonged use can lead to severe hepatic damage [3]. AILI is the most prevalent cause of DILI, accounting for a significant proportion of ALF cases [4]. N-acetylcysteine (NAC) is the only treatment for APAP overdose. However, NAC has limited efficacy and severe side effects [5,6]. Consequently, innovative approaches to treat AILI are urgently needed.

Hepatocyte damage represents a central mechanism in inflammation and disease progression in a variety of acute and chronic liver disorders [7]. Recent studies have shown that cellular pyroptosis is the predominant mode of cell death during the progression of AILI [8]. The activation of the NOD-, LRR-, and pyrin domain-containing protein 3 (NLRP3) inflammasome plays a critical role in pyroptosis, leading to the activation of cysteine–aspartic protease 1 (caspase-1) [9]. This activation results in the release of pro-inflammatory cytokines, ultimately culminating in cell death. Furthermore, caspase-1 cleaves gasdermin D (GSDMD) to produce its N-terminal fragment, which forms pores in the cell membrane, thereby inducing pyroptosis. During pyroptosis, pro-inflammatory cytokines will release from damaged hepatocytes, including interleukin-1β (IL-1β), tumor necrosis factor-alpha (TNF-α), interleukin-6 (IL-6), interleukin-18 (IL-18), and high-mobility group box 1 (HMGB1) [10,11]. These cytokines further activate immune cells, exacerbating hepatocyte pyroptosis and contributing to liver injury.

Neutrophils are the most abundant white blood cells and are closely related to inflammation [12]. Neutrophils migrate to the liver during the progression of AILI, leading to the formation of neutrophil extracellular traps (NETs) [13]. NETs are a complex extracellular structure composed of chromatin and granular proteins, including citrullinated histone H3 (CitH3), myeloperoxidase (MPO), neutrophil elastase (NE), peptidyl arginine deiminase 4 (PADI4), and DNA filaments [14]. It has been demonstrated that NETs can further aggravate the progression of AILI [15]. However, the relationship between hepatocyte pyroptosis and NETs in AILI remains unclear.

Recent findings indicate that HMGB1 can exacerbate liver inflammation and induce the formation of NETs during hepatocyte pyroptosis [16]. Hepatocyte pyroptosis can promote the release of HMGB1 from hepatocytes. Since HMGB1 can aggravate the formation of NETs, targeting this crosstalk can offer a novel therapeutic target for the treatment of AILI and other liver diseases. Exploring the mechanism of AILI progression is essential for developing treatment strategies for AILI.

Paeoniflorin (PF) is the main bioactive ingredient derived from *Paeonia lactiflora* Pallas. PF exhibits diverse pharmacological properties, including anti-inflammatory, antiviral, antibacterial, antioxidant, and immunomodulatory effects [17,18,19,20,21]. Increasing evidence suggests that PF can effectively ameliorate various liver conditions, including cholestasis, hepatitis, non-alcoholic fatty liver disease, and DILI. Recently, it has been reported that PF can treat liver injury through a variety of mechanisms, including regulating the CYP2E1/JNK and LKB1/AMPK signaling pathways to affect bile acid enterohepatic circulation, and the insulin signaling pathway to protect against liver injury [22,23,24,25]. Researchers have reported that that low doses of PF (below 100 mg/kg) can effectively treat liver injury in vivo [26,27]. Our preliminary experiments also confirmed that PF at doses ranging from 20 mg/kg to 100 mg/kg could significantly attenuate AILI. However, the underlying mechanisms through which PF attenuates AILI remain unclear. In this study, we investigated the effect of PF on AILI treatment and found that PF interferes with the crosstalk between hepatocyte pyroptosis and NETs by regulating HMGB1.

## 2. Results

### 2.1. PF Attenuates AILI in Mice

**Figure 1:**
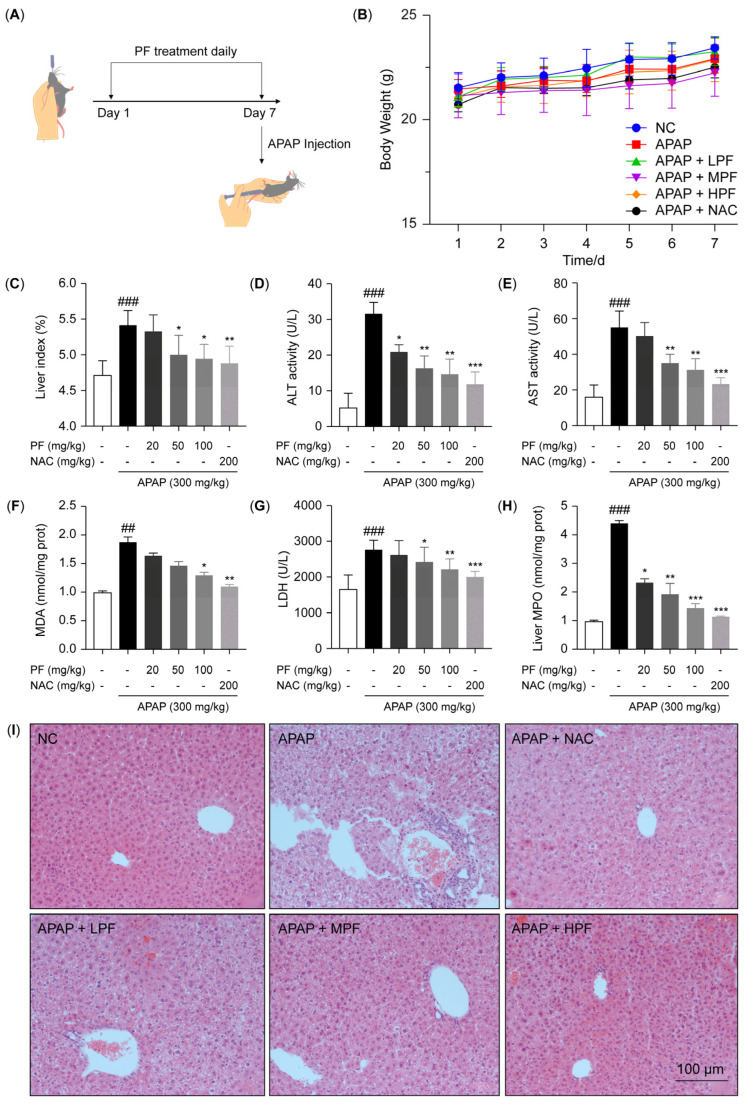
PF attenuates AILI in mice. (**A**) Schematic diagram of drug administration to animals (n = 6); (**B**) body weights of mice during administration; (**C**) liver index measurements; (**D**–**G**) serum concentrations of ALT, AST, LDH, and MDA; (**H**) MPO levels in liver tissues of mice; (**I**) H&E staining images. The magnification used for the H&E staining images was ×100, with the scale bars representing 100 μm. All experimental data are presented as the mean ± SD. ## *p* < 0.01 and ### *p <* 0.001 vs. the NC group; * *p* < 0.05, ** *p* < 0.01, and *** *p* < 0.001 vs. the APAP group.

The effect of PF on AILI in mice was assessed using the intragastric administration protocol depicted in Figure 1A. Throughout the entire in vivo experiment, there were no significant differences in body weight in the experimental groups compared to the negative control (NC) group. This indicated that the administration of PF and NAC caused no significant systemic toxicity in mice (Figure 1B). The treatment with APAP significantly increased the liver index, while the administration of PF and NAC mitigated this increase (Figure 1C). Histopathological evaluations, hepatic MPO levels, along with serum levels of alanine transaminase (ALT), aspartate transaminase (AST), lactate dehydrogenase (LDH), and malondialdehyde (MDA), were extensively utilized to evaluate the potential protective effect of the drugs on the AILI. Compared to the negative control (NC) group, the APAP treatment increased the serum levels of AST, ALT, MDA, and LDH. However, PF and NAC administration significantly reduced these levels (Figure 1D–G). In comparison to the NC group, the hepatic MPO levels were elevated in the APAP-treated group. However, PF and NAC administration markedly reduced the MPO levels (Figure 1H). Hematoxylin and eosin (H&E) staining of liver tissues revealed that the APAP treatment resulted in an irregular hepatocyte arrangement and cytoplasmic vacuolization, accompanied by significant inflammatory cell infiltration, which were all significantly mitigated by treatment with PF and NAC (Figure 1I). These findings indicate that PF can effectively alleviate AILI.

### 2.2. PF Attenuates APAP-Induced Hepatocyte Inflammation and Pyroptosis

**Figure 2:**
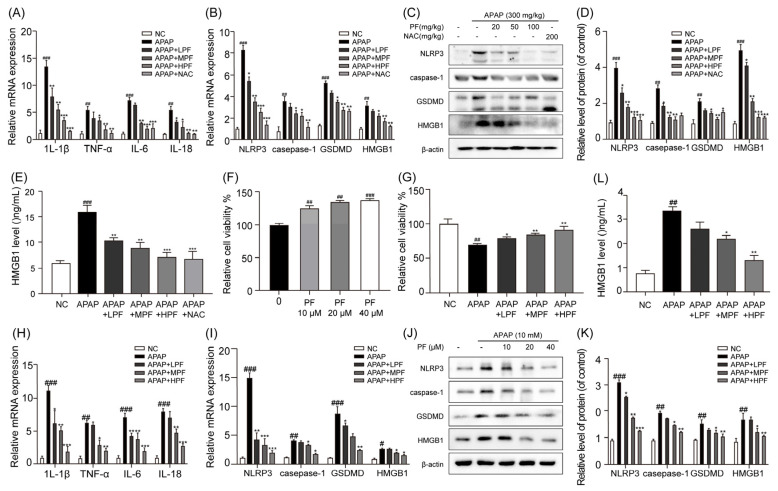
PF attenuates APAP-induced hepatocyte inflammation and pyroptosis. (**A**) The mRNA expression levels of *IL-1β*, *TNF-α*, *IL-6*, and *IL-18* in the liver of the mice were quantified using the quantitative real-time polymerase chain reaction (qRT-PCR). (**B**) The mRNA expression levels of *NLRP3*, *caspase-1*, *GSDMD*, and *HMGB1* in the livers of mice were measured via qRT-PCR. (**C**,**D**) The protein levels of NLRP3, caspase-1, GSDMD, and HMGB1 in the livers of mice were assessed using Western blotting (WB) analysis and are reported relative to β-actin levels. (**E**) The levels of HMGB1 in the serum of mice were quantified using enzyme-linked immunosorbent assays (ELISAs). (**F**) HepG2 cells were treated with various concentrations of PF (0, 10, 20, and 40 μM) for 24 h, and then the cell viability was assessed using the thiazolyl blue tetrazolium bromide (MTT) assay (n = 3). (**G**) HepG2 cells were pretreated with various concentrations of PF (0, 10, 20, and 40 μM) for 24 h and subsequently treated with APAP (10 mM) for 6 h, and then the cell viability was assessed using the MTT assay (n = 3). (**H**) The mRNA expression levels of *IL-1β*, *TNF-α*, *IL-6*, and *IL-18* in HepG2 cells were quantified using qRT-PCR. (**I**) The mRNA expression levels of *NLRP3*, *caspase-1*, *GSDMD*, and *HMGB1* in the HepG2 cells were quantified using qRT-PCR. (**J**,**K**) The protein levels of NLRP3, caspase-1, GSDMD, and HMGB1 in the HepG2 cells were assessed using WB analysis, and are reported relative to β-actin levels. (**L**) The concentration of HMGB1 in the culture medium of the HepG2 cells was quantified using ELISA. All experimental data are presented as the mean ± SD. ## *p* < 0.01 and ### *p* < 0.001 vs. the NC group; * *p*< 0.05, ** *p*< 0.01, and *** *p*< 0.001 vs. the APAP group.

Pyroptosis is an inflammatory mode of cell death that leads to the massive release of HMGB1 from hepatocytes [28]. Figure 2A shows that the mRNA expression of the inflammatory cytokines *IL-1β*, *TNF-α*, *IL-6*, and *IL-18* significantly increased in the liver of the mice treated with APAP, in contrast to the levels observed in the NC group. The administration of PF and NAC significantly inhibited the release of these inflammatory mediators. Compared with the NC group, APAP intervention increased the mRNA and protein expression levels of NLRP3, caspase-1, and GSDMD in the liver of the mice; these expression levels were reduced by PF and NAC administration (Figure 2B–D). In comparison to the NC group, the mRNA and protein expression levels of HMGB1 were elevated after APAP treatment, while PF and NAC significantly attenuated these levels. The same was observed for the serum HMGB1 levels (Figure 2B–E). These findings indicate that PF can effectively alleviate the inflammation and pyroptosis induced by APAP.

Human hepatoma G2 (HepG2) cells and alpha mouse liver 12 (AML-12) cells are widely used as in vitro models to investigate the mechanisms underlying AILI [29,30]. HepG2 and AML-12 cells were used to further explore the effect of PF on hepatocyte pyroptosis. PF significantly enhanced the cell viability in both HepG2 and AML-12 cells (Figure 2F and Figure S2B). Compared with the NC group, the APAP treatment decreased HepG2 and AML-12 cell viability, which was reversed by PF (Figure 2G and Figure S2C). The mRNA levels of the inflammatory factors *IL-1β*, *TNF-α*, *IL-6*, and *IL-18* in both the HepG2 and AML-12 cells were increased by APAP. However, the PF treatment markedly reduced these levels (Figure 2H and Figure S2D). Compared to the APAP group, the PF treatment reduced the mRNA and protein expression levels of NLRP3, caspase-1, and GSDMD (Figure 2I–K and Figure S2E). In addition, we investigate the effects of PF on the NLRP3 inflammasome, one of the upstream activators of pyroptosis. The activation of NLRP3 leads to the activation of caspase-1 and GSDMD, which, in turn, induces pyroptosis [31,32]. Therefore, we speculated that PF acts upstream of inflammasome activation, but the specific pathway requires further investigation. In HepG2 cells, PF effectively inhibited the mRNA and protein levels of HMGB1, both intracellularly and extracellularly, following APAP stimulation (Figure 2I–L). These findings demonstrate that PF can effectively alleviate APAP-induced hepatocyte inflammation and pyroptosis.

### 2.3. PF Inhibits the Formation of NETs Induced by APAP

**Figure 3:**
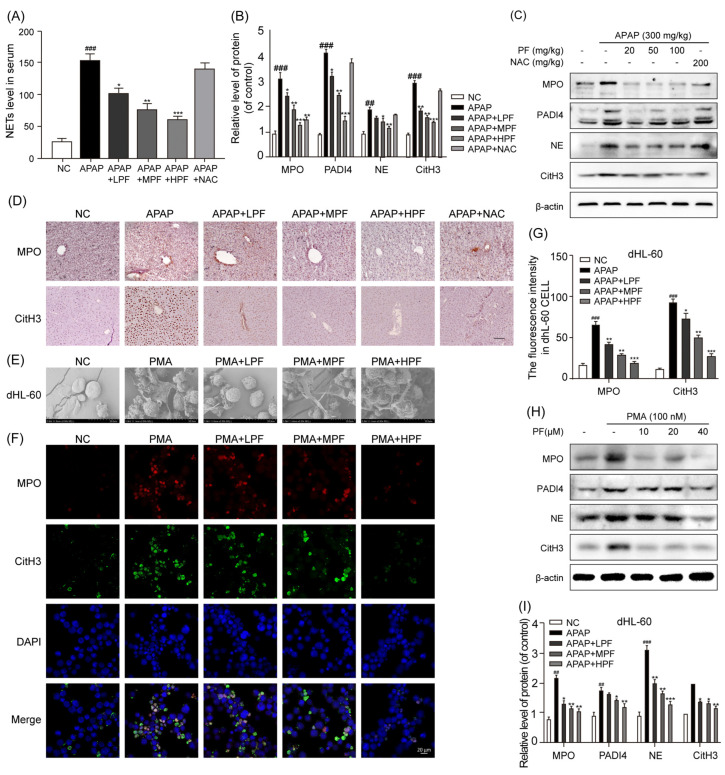
PF inhibits the APAP-induced formation of NETs. (**A**) NETs in the serum were quantified using an ELISA for NE–DNA complexes. (**B**,**C**) The protein levels of MPO, PADI4, NE, and CitH3 in the mouse liver were assessed using WB analysis and are reported relative to β-actin levels. (**D**) To evaluate the distribution of MPO and CitH3, the positive areas for MPO and CitH3 in liver sections were assessed using IHC staining. The magnification in the images is ×200. (**E**) The surface characterization of the dHL-60 cells was performed using FESEM. The magnification used was ×2500, with the scale bars representing 20 μm. (**F**,**G**) A cellular immunofluorescence analysis was conducted to assess the protein expression of MPO and CitH3 in the dHL-60 cells (n = 3). The magnification used was ×400, with the scale bars representing 20 μm. (**H**,**I**) The protein levels of MPO, PADI4, NE, and CitH3 in the dHL-60 cells were assessed using WB analysis, and are reported relative to β-actin levels. All experimental data are presented as the mean ± SD. ## *p* < 0.01 and ### *p* < 0.001 vs. the NC group; * *p* < 0.05, ** *p* < 0.01, and *** *p* < 0.001 vs. the APAP group or PMA group.

The predominant inflammatory cell infiltrating the liver in AILI is neutrophils [33,34]. Compared with the NC group, the serum levels of NE-DNA complexes (a NETs marker) were significantly increased in the APAP-treated group. The PF treatment significantly reduced the formation of NETs in the serum, whereas NAC did not exhibit a comparable effect (Figure 3A). The administration of PF decreased the protein expression levels of MPO, PADI4, NE, and CitH3 in the liver of the mice treated with APAP. However, NAC was less effective than PF in inhibiting NETs formation (Figure 3B,C). The immunohistochemistry (IHC) staining analysis of liver tissues revealed that the protein expression levels of MPO and CitH3 were elevated in the APAP-treated group compared with the NC group. The administration of PF and NAC significantly reduced the expression of these proteins (Figure 3D). Collectively, these findings suggest that PF can inhibit the formation of NETs in AILI.

We further assessed the impact of PF on NETs formation in PMA-stimulated, neutrophil-like, differentiated HL-60 (dHL-60) cells. Compared with the NC group, the PMA stimulation induced morphological changes in the dHL-60 cells, accompanied by the release of cellular contents, which could be inhibited by PF (Figure 3E). We observed a significant increase in the fluorescent intensity of MPO and CitH3 proteins in the dHL-60 cells in the PMA-treated group compared to the NC group. The protein expression of MPO, PADI4, NE, and CitH3 in the dHL-60 cells was also significantly increased after the PMA treatment. In contrast, the expression levels of these proteins were significantly reduced in the PF treatment group (Figure 3F–I). Collectively, these findings suggest that PF effectively inhibits PMA-induced NETs formation.

### 2.4. PF Intervenes the Crosstalk Between Hepatocyte Pyroptosis and NETs In Vitro

**Figure 4:**
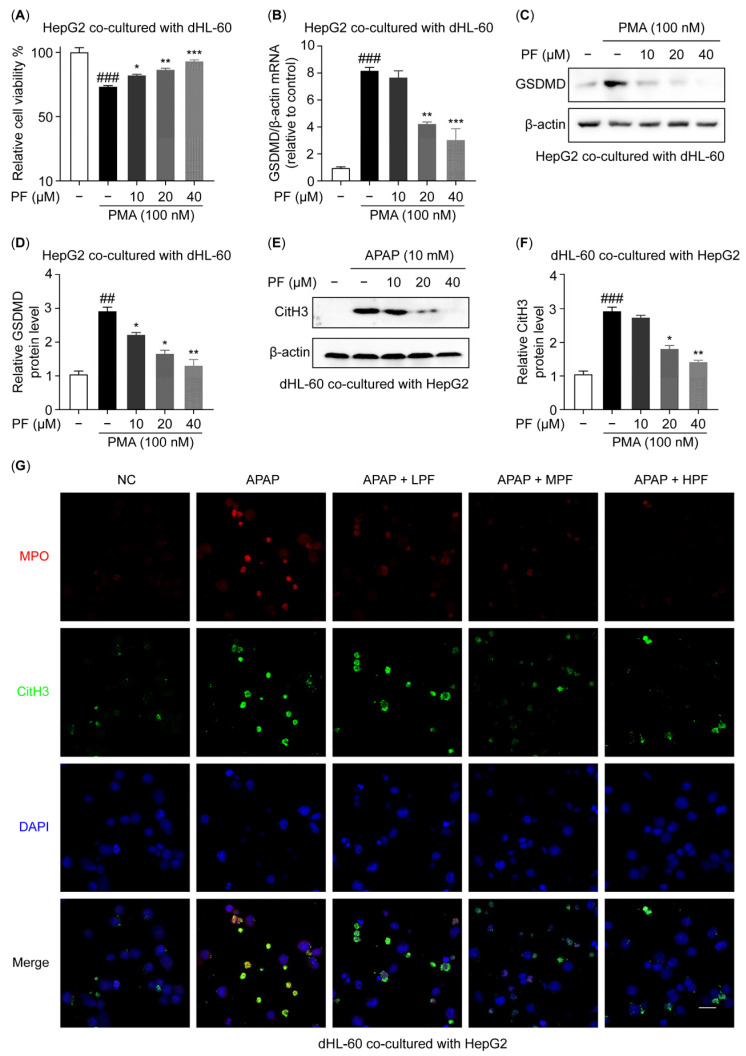
PF intervenes the crosstalk between hepatocyte pyroptosis and NETs. (**A**) HepG2 cells in the co-culture were treated with PMA (100 nM) for 4 h, followed by treatment with various concentrations of PF (0, 10, 20, and 40 μM) for 24 h; subsequently, cell viability was assessed using the MTT assay (n = 3). (**B**) The mRNA expression level of GSDMD in the HepG2 cells in the co-culture was quantified using qRT-PCR. (**C**,**D**) The protein level of GSDMD in the HepG2 cells in the co-culture was assessed by WB analysis and reported relative to β-actin levels. (**E**,**F**) The protein levels of CitH3 in the dHL-60 cells in the co-culture were assessed using WB analysis and are reported relative to β-actin levels. (**G**) A cellular immunofluorescence analysis was performed to evaluate the expression of MPO and CitH3 in the dHL-60 cells in the co-culture (n = 3). The magnification used was ×400. All experimental data are presented as the mean ± SD. ## *p* < 0.01 and ### *p* < 0.001 vs. the NC group; * *p* < 0.05, ** *p* < 0.01, and *** *p* < 0.001 vs. the APAP group or PMA group.

To explore the effect of PF on the crosstalk between hepatocyte pyroptosis and NETs, co-culture experiments using neutrophils and hepatocytes were conducted. Firstly, to elucidate the effects of NETs formation on hepatocyte pyroptosis, PMA-stimulated dHL-60 cells were co-cultured with HepG2 cells. As shown in Figure 4A, in the co-culture system, PMA-induced NETs reduce the viability of HepG2 cells, while treatment with PF markedly restores cell viability. Compared with the NC group, the expression levels of the mRNA and protein of GSDMD were elevated in HepG2 cells upon the formation of NETs (Figure 4B–D). These results indicate that PF attenuates NETs-induced pyroptosis in hepatocytes. Secondly, to investigate the influence of hepatocyte pyroptosis on NETs production from neutrophils, APAP-treated HepG2 cells were co-cultured with dHL-60 cells. In comparison to the NC group, hepatocyte pyroptosis markedly enhanced the expression levels of MPO and CitH3 in the dHL-60 cells, while PF attenuated the expression levels of these proteins (Figure 4E–G). These findings suggest that PF has the potential to inhibit the formation of NETs stimulated by APAP-induced hepatocyte pyroptosis. Collectively, PF can intervene in the crosstalk between hepatocyte pyroptosis and NETs.

### 2.5. PF Attenuates AILI in Mice by Intervening the Crosstalk Between Hepatocyte Pyroptosis and NETs

**Figure 5:**
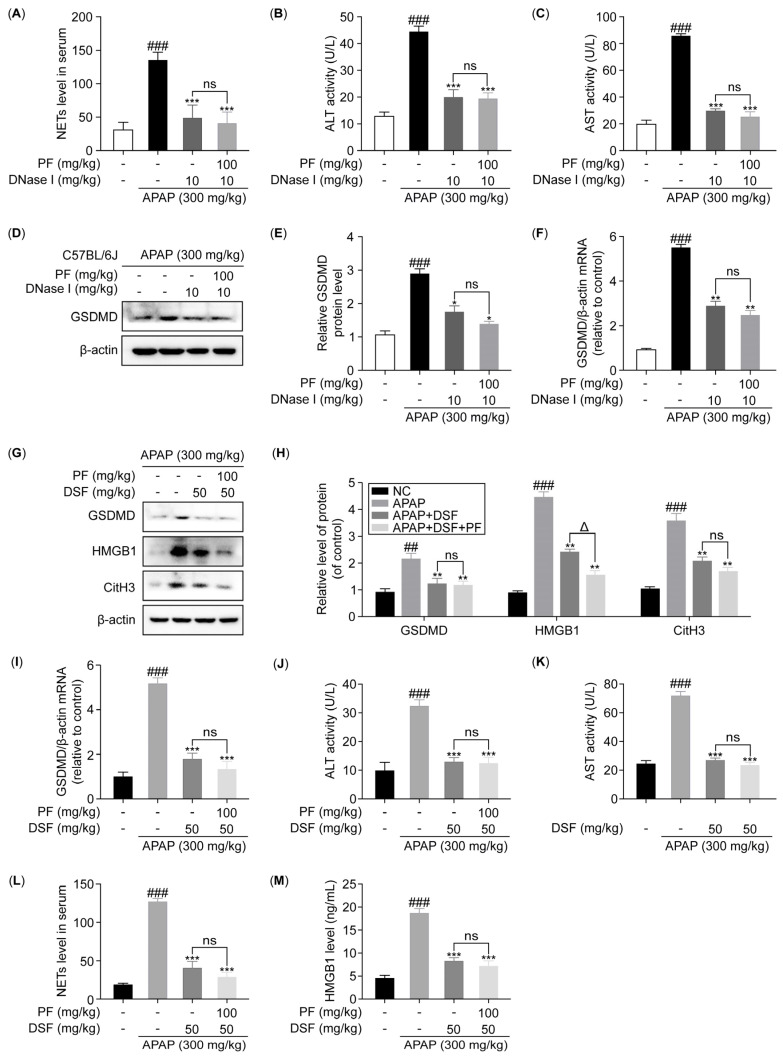
PF attenuates AILI in mice by intervening the crosstalk between hepatocyte pyroptosis and NETs. (**A**) The levels of NETs in the serum of the neutropenic mice were quantified using ELISAs. (**B**,**C**) The ALT and AST levels in the serum of the neutropenic mice. (**D**,**E**) The protein levels of GSDMD in the liver of the neutropenic mice were determined using WB analysis and are reported relative to β-actin levels. (**F**) The mRNA expression level of GSDMD in the liver of the neutropenic mice was quantified via qRT-PCR. (**G**,**H**) The protein levels of GSDMD, HMGB1, and CitH3 in the liver of the pyroptosis inhibitor-treated mice were assessed using WB analysis and are reported relative to β-actin levels. (**I**) The mRNA expression level of GSDMD in the liver of the pyroptosis inhibitor-treated mice was quantified via qRT-PCR. (**J**,**K**) The serum levels of ALT and AST in the pyroptosis inhibitor-treated mice. (**L**,**M**) The levels of NETs and HMGB1 in the serum of the pyroptosis inhibitor-treated mice were measured using ELISA. All experimental data are presented as the mean ± SD. ## *p* < 0.01 and ### *p* < 0.001 vs. the NC group, * *p* < 0.05, ** *p* < 0.01, and *** *p* < 0.001 vs. the APAP or PMA group; ^Δ^ *p* < 0.05, ns *p* > 0.05 vs. the APAP + DNase I or APAP + DSF group.

To further validate the above mentioned in vitro conclusions, we performed in vivo studies. Initially, the mice were injected with Deoxyribonuclease I (DNase I) into their tail veins, and the levels of NETs were found to be significantly reduced in the serum (Figure 5A). The serum levels of ALT and AST were reduced in the mice after treatment with the NETs inhibitor DNase I. Notably, the combination of DNase I and PF did not further reduce the levels of ALT and AST. When comparing the use of PF in combination with DNase I to DNase I alone, the reduction in ALT levels decreased from 46.857% to 44.347%. Similarly, the decrease in AST levels was reduced from 39.927% to 33.419% when PF was added to the DNase I treatment (Figure 5B,C). These results indicate that the mitigating effect of PF on AILI is mediated by its effects on NETs. The elevated expression levels of GSDMD mRNA and protein in APAP-treated mouse liver were significantly inhibited by DNase I. The combined treatment of DNase I and PF had no significant effect on the GSDMD levels (Figure 5D–F). These results suggest that PF inhibits hepatocyte pyroptosis by intervening in the formation of NETs.

Given that disulfiram (DSF) can inhibit pyroptosis by preventing GSDMD pore formation, we subsequently treated the mice with DSF. As shown in Figure 5G–I, the protein and mRNA expression levels of GSDMD were significantly suppressed by DSF. Compared with the APAP group, the serum ALT and AST levels were significantly lower in the DSF-treated group. The combination of DSF and PF did not lead to further reductions in the ALT and AST levels. Compared with DSF alone, PF combined with DSF reduced the ALT and AST levels from 46.857% and 39.927% to 45.347% and 36.419%, respectively (Figure 5J,K). The results show that PF alleviates the AILI by inhibiting hepatocyte pyroptosis. Compared with the APAP group, when the mice were administered DSF, the levels of HMGB1 in the liver and serum were also significantly reduced (Figure 5G,H,M). Similarly, DSF inhibited CitH3 protein levels in the liver and NETs levels in the serum of the APAP-treated mice. The combination of DSF and PF did not lead to further changes in the levels of CitH3 and NETs (Figure 5G,H,L). These results suggest that PF can interfere with NETs’ formation by inhibiting hepatocyte pyroptosis.

In summary, these results indicat that hepatocyte pyroptosis and NETs both promote liver damage in AILI, and PF can alleviate AILI by intervening in the crosstalk between hepatocyte pyroptosis and NETs.

## 3. Discussion

DILI refers to toxic liver damage caused by drugs and their metabolites. DILI is responsible for around 15% of acute liver failure cases in the United States and Europe, and its incidence is rising [35]. In about 80% of patients, the symptoms resolve after the medication is discontinued, but some patients may continue to develop chronic or acute liver failure. The treatment options for DILI are relatively limited, typically involving the use of hepatocyte repair or protective agents, with liver transplantation considered in severe cases [1]. APAP overdose is a leading cause of DILI. APAP is a commonly used analgesic drug that is safe and effective at the recommended doses. However, excessive use of APAP may lead to severe liver injury. The mechanism of AILI is mainly related to the direct toxic effect of its metabolites on hepatocytes and the increase in oxidative stress [36]. Administering APAP to mice is a widely used method for studying DILI. It can mimic the human response to APAP, and a sublethal dose of 300 mg/kg can effectively induce liver injury, making it convenient to observe the effect of drug interventions [37]. The HepG2 cell line originates from human hepatocytes and possesses excellent biological characteristics, and it is widely used in in vitro studies of DILI [30]. Therefore, in the present study, APAP-induced mice and HepG2 cells were used to establish AILI models to investigate the alleviation effect of PF on AILI and its mechanism. Previous studies have confirmed that PF has a significant protective effect against AILI. Our study also showed that PF was able to significantly reduce the liver index of AILI mice and reduce the levels of ALT, AST, MDA, and LDH in the serum of mice, while reducing the level of MPO in the liver of mice. In addition, PF was able to reverse the reduced viability of HepG2 cells caused by APAP. However, the specific mechanism of PF in alleviating AILI still needs to be further explored in order to fully understand its mechanism of action and clinical application potential. PF is the active ingredient extracted from the roots of ranunculaceae *Paeonia lactiflora* Pall and *Paeonia veitchii* Lynch. PF possesses notable pharmacological properties, including antioxidant, anti-inflammatory, analgesic, and immunomodulatory actions, with a particular emphasis on hepatoprotection. A number of studies have confirmed the protective effect of PF on AILI [19,20,38,39,40]. Studies have shown that PF initiates autophagy by regulating the MAPK/mTOR signaling pathway, thereby alleviating AILI [26]. In addition, PF also reduces the levels of mitochondrial metabolic enzymes by inhibiting the JNK/CYP2E1 signaling pathway, which effectively alleviates the hepatocyte injury caused by APAP [22].

In the process of liver injury, inflammation is a key pathological feature, which is not only a protective response against hepatocyte injury, but also closely related to pyroptosis. Pyroptosis is a type of programmed cell death that is mediated by the inflammasome and is characterized by the breakdown of cell membranes and the release of proinflammatory factors, such as IL-1β, TNF-α, IL-6, and IL-18, which further recruit immune cells and exacerbate local and systemic inflammation [41]. HMGB1 plays a key role in pyroptosis; it can be actively and passively released from damaged or necrotic cells [42]. The released HMGB1 binds to receptors, such as RAGE and TLR4, recruits immune cells, and enhances the inflammatory response [43]. In addition, the release of HMGB1 is related to cell membrane rupture, and the release of cell contents during pyroptosis further promotes the excretion of HMGB1, exacerbating local inflammation [44]. In liver injury, HMGB1 acts as an important pro-inflammatory factor, which aggravates acute and chronic liver injury by activating endothelial cells and promoting the recruitment of neutrophils and macrophages [45]. Therefore, regulating the release of HMGB1 and its signaling pathway is regarded as an important strategy for the treatment of liver disease. In this study, we found that PF significantly reduced the mRNA expression of inflammation- and pyroptosis-related factors and pyroptosis-related proteins in the AILI mice and HepG2 cells. Meanwhile, PF decreased the HMGB1 levels in the serum and liver of the AILI mice and decreased HMGB1 release from the HepG2 cells after APAP treatment. These results suggest a potential therapeutic effect of PF in reducing liver injury, but the specific mechanisms need to be further investigated.

DSF is an approved drug for the treatment of alcohol addiction and has been extensively studied as a pyroptosis inhibitor in recent years. However, the data on the clinical use of DSF for liver protection are relatively limited, and its low bioavailability may affect its efficacy. In addition, the mechanism of action of disulfiram is not fully understood, and it has side effects and tolerability issues, such as skin rashes. In contrast, clinically, PF shows promising therapeutic potential for diseases such as Alzheimer’s disease, Parkinson’s disease, and rheumatoid arthritis [46]. In addition, PF has been investigated for use in areas such as cardiovascular disease and diabetes, showing its broad applicability [47,48]. As a traditional Chinese medicine monomer with high safety and low toxicity, paeoniflorin is expected to become a new pyroptosis inhibitor.

NETs are intricate formations created by neutrophils when they react to certain triggers, primarily serving the function of trapping and eliminating infectious agents. The main components of NETs are DNA, histones, and MPO, which together form a network structure with DNA as the skeleton. NETs formation process is called NETosis and includes neutrophil activation, chromatin depolymerization, and cell membrane rupture [49,50]. NETs are closely related to inflammatory responses, and they release proinflammatory factors to aggravate local and systemic inflammation. HMGB1 is an important molecule released by NETs, which can recruit and activate more neutrophils, thereby aggravating the inflammatory response [51]. We showed that the activation of hepatocyte pyroptosis in AILI is accompanied by the release of large amounts of HMGB1. In the drug-induced liver injury model, the expression of NETs significantly increased, which was related to the degree of liver inflammation and injury. The findings of our research reveal a marked upregulation of NETs-associated proteins, including MPO and CitH3, in the AILI mouse model, suggesting a role for NETs in the onset of AILI. However, PF could effectively inhibit the expression of NETs-related proteins in AILI mice and reduce the formation of NETs in human leukemia HL-60 cells under PMA stimulation. These results suggest that PF exerts a potential protective effect on liver injury by regulating NETs formation.

In the process of liver inflammation, there is a complex relationship between pyroptosis and NETs. Both pyroptosis and NETs promote each other in the inflammatory response: NETs not only capture pathogens, but also release proinflammatory factors that exacerbate local inflammation and induce pyroptosis [52]. To investigate the effect of NETs on pyroptosis, we used the neutrophil inhibitor DNase I and the pyroptosis inhibitor DSF. DNase I is an enzyme capable of efficiently degrading extracellular DNA and interferes with NETosis by hydrolytically cleaving DNA strands to destroy the DNA skeleton required for NETs [53,54]. In the AILI mouse model, the expression of key pyroptosis-related proteins was significantly inhibited after the DNase I treatment, suggesting that NETs play an important role in promoting pyroptosis. At the same time, as a safe clinical drug, recent studies have found that DSF can inhibit the membrane puncturing effect of the activated pyroptosis executive protein GSDMD by covalently modifying the specific site of DSF, thereby exerting an anti-pyroptosis effect [55,56]. In the AILI mice, DSF administration significantly reduced the formation of NETs in the liver and serum, while the efficacy of PF in alleviating AILI was also reduced, supporting the interdependence between pyroptosis and NETs. In order to further explore the relationship between HepG2 and dHL-60 cells, we carried out co-culture experiments. When the APAP-stimulated HepG2 cells underwent pyroptosis, the dHL-60 cells formed NETs, and PF was able to effectively inhibit this process. Similarly, the HepG2 cells also underwent pyroptosis when the dHL-60 cells were stimulated by PMA to form NETs, which was similarly inhibited by PF. These findings indicate a substantial interaction between pyroptosis and NETs in the development of AILI, and that PF can mitigate AILI by disrupting this interplay.

In this study, we revealed that APAP induces liver injury by promoting hepatocyte pyroptosis and NETs formation, suggesting that targeting the interaction between these two factors may be an effective therapeutic approach. Second, we clarified the role of PF in alleviating AILI by regulating the interaction between hepatocyte pyroptosis and NETs. These findings offer new insights into the pharmacological mechanism of PF in treating AILI and provide a scientific basis for the development of PF as a clinical drug for the treatment of AILI. This study contributes to a deeper understanding of the relationship between pyroptosis and NETs, thereby leading to the development of more effective interventions for AILI. In summary, as a natural compound with a broad range of pharmacological activities, PF brings new hope to the treatment of liver diseases.

## 4. Materials and Methods

### 4.1. Reagents

The PF was purchased from MedChemExpress (Monmouth Junction, NJ, USA); it had a purity of 98.38%, which met the excellent quality standard for the pharmacological research. All other materials and reagents are listed in Supplementary Table S1.

### 4.2. Animal Experiments

Male *C57BL/6J* mice, aged 4–6 weeks, were obtained from the Shanghai Slaughter Laboratory Animal Co. (Shanghai, China). The mice were housed in a well-ventilated environment maintained in a temperature range of 18–25 °C and relative humidity of 40–70%, with a 12 h light/dark cycle. After acclimatization for one day, the mice were treated with three distinct experimental protocols. (1) AILI mouse model: The mice were randomly divided into an NC group, APAP (300 mg/kg) group, APAP (300 mg/kg) + low-dose PF group (LPF, 20 mg/kg), APAP (300 mg/kg) + medium-dose PF group (MPF, 50 mg/kg), APAP (300 mg/kg) + high-dose PF group (HPF, 100 mg/kg), and APAP (300 mg/kg) + NAC (200 mg/kg) group (n = 6 mice/group). The NC and APAP groups received oral saline for seven days; the NAC group received oral NAC at a dosage of 200 mg/kg for seven days; and the PF group received oral PF at dosages of 20, 50, and 100 mg/kg over the same duration. For the dosing of these agents, we referred to the literature [26,27,57,58]. Except for the NC group, all mice received a single intraperitoneal injection of APAP (300 mg/kg) two hours prior to the administration of the final dose. (2) NETs-depletion mouse model: This model was designed to examine the effect of NETs on hepatocyte pyroptosis in AILI. The mice were randomly divided into an NC group, APAP group, DNase I (to deplete NETs) group, and PF + DNase I group (n = 6 mice/group). The PF + DNase I group was administered PF (100 mg/kg) orally for seven days, while the other groups received saline. For the dosing of DNase I, we referred to a previous study [54]. One hour after the APAP injection, the mice in both the DNase I and PF + DNase I groups were administered DNase I (10 mg/kg) via the tail vein, while the NC group received an equivalent volume of sterile water. (3) Pyroptosis inhibitor mouse model: This model was established to assess the role of pyroptosis in NETs formation in AILI. The mice were randomly divided into four groups (n = 6): an NC group, APAP group, DSF (can inhibit pyroptosis) group, and PF + DSF group (n = 6 mice/group). The PF + DSF group received oral PF (100 mg/kg) for seven days, while the other groups received saline. The mice in both the DSF and PF + DSF groups were administered DSF intraperitoneally at a dosage of 50 mg/kg four hours and twenty-four hours prior to APAP administration. The dosing of DSF followed that in a previous study [59]. In all the groups, the mice were fasted for twelve hours before the APAP injection, but had unrestricted access to water. Six hours following the APAP injection, the mice were euthanized, and blood and liver samples were collected for the subsequent analyses.

### 4.3. Plasma and Liver Biochemical Measures

The liver weight and body weight of the mice were determined, and the liver index was calculated using the following formula: liver index (%) = (liver weight [g]/body weight [g]) × 100%. Blood samples were collected from the mice and allowed to stand at 37 °C for 30 min. Subsequently, the blood was centrifuged at 3000 rpm for 15 min at 4 °C, and the serum was collected and stored at −80 °C until further analysis. The serum levels of ALT, AST, LDH, and MDA were measured using commercially available assay kits following the manufacturer’s instructions. Following blood collection, the mice were euthanized via cervical dislocation, and their livers were excised, immediately frozen in liquid nitrogen, and subsequently stored at −80 °C. The MPO levels in the liver were quantified using a commercial assay kit following the manufacturer’s instructions.

### 4.4. Histological Analysis

Mouse liver tissues were fixed in 10% neutral formalin. The fixed tissue blocks were dehydrated by soaking in 30%, 50%, 70%, 95%, and absolute ethanol solutions in turn. They were then cleared with xylene and embedded in paraffin. The paraffin-embedded tissues were cut into 5 µm thick sections using a microtome to facilitate the subsequent staining and microscopic observation. Hematoxylin and eosin (H&E) staining was applied. Hematoxylin was used to stain the nuclei, while eosin was used to stain the cytoplasm and connective tissues. This method is widely used to observe the pathological changes in liver cell injury, necrosis, and fibrosis [60]. The liver tissue sections were stained with H&E using a commercial assay kit according to the manufacturer’s instructions. Observations were performed with a Zeiss Axio Zeiss Scope A1 inverted fluorescence microscope (Carl Zeiss, Inc., Oberkochen, Germany) at a magnification of 100× to allow for the clear visualization of the nuclear and cytoplasmic staining.

### 4.5. ELISA

ELISAs were performed according to the manufacturer’s instructions to detect the levels of HMGB1 and NETs in the serum and cell culture medium.

### 4.6. IHC Staining

The paraffin-embedded liver tissue sections were deparaffinized with xylene and dehydrated using a gradient of ethanol. Endogenous peroxidase activity was blocked with 3% hydrogen peroxide (H_2_O_2_), and the sections were sealed with 5% bovine serum albumin. The tissue sections were incubated overnight at 4 °C with primary antibodies against MPO (dilution: 1:1000) and CitH3 (dilution: 1:500). After washing three times with phosphate-buffered saline (PBS), the sections were incubated with a goat anti-rabbit secondary antibody for 30 min at 37 °C. Subsequently, the diaminobenzidine color development solution was added dropwise. The sections were then counterstained with hematoxylin, mounted with neutral balsam, and observed under a microscope for imaging.

### 4.7. Cell Culture

The HepG2, AML-12, and human promyelocytic leukemia (HL-60) cells were purchased from the Shanghai Institute of Biochemistry and Cell Biology, Chinese Academy of Sciences (Shanghai, China). The HepG2 and AML-12 cells were cultured in DMEM supplemented with 10% fetal bovine serum (FBS), 100 IU/mL penicillin, and 100 μg/mL streptomycin. The HL-60 cells were maintained in RPMI 1640 medium supplemented with 10% FBS. The cells were incubated in a humid atmosphere containing 5% CO_2_ at 37 °C and passaged according to the recommended procedures established by the ATCC.

### 4.8. Cell Viability Assay

The HepG2 cells and AML-12 cells were initially plated in a 96-well cell culture plate at a density of 1.5 × 10^4^ cells/well. Part A: To explore the effect of APAP on HepG2 and AML-12 cells, the HepG2 cells were exposed to 10 mM APAP for 6, 12, and 24 h, while the AML-12 cells were exposed to 2.5 mM, 5 mM, and 10 mM APAP for 4 and 6 h (Supplementary Figures S1A and S2A). For the subsequent experiments, the HepG2 cells were stimulated with 10 mM APAP for 6 h, while the AML-12 cells were stimulated with 5 mM APAP for 4 h [61,62]. Part B: To explore the effect of PF on HepG2 and AML-12 cells, these cells were treated with LPF (10 μM), MPF (20 μM), and HPF (40 μM) for 24 h. Part C: To investigate the effect of PF on the damage caused by APAP to HepG2 and AML-12 cells, the cells were treated with PF for 24 h, followed by exposure to APAP.

The HL-60 cells were induced using 10 μM all-trans retinoic acid (ATRA) for seven days to become dHL-60 cells. Subsequently, the dHL-60 cells were stimulated with 100 nM phorbol 12-myristate 13-acetate (PMA) for four hours to generate NETs [63]. Cell viability was assessed using the MTT assay. Briefly, MTT (5 mg/mL, 20 μL per well) was added to the cells and incubated for 4 h at 37 °C. Afterward, the supernatant was removed, and dimethyl sulfoxide (100 μL per well) was added before incubating the plate at 37 °C for an additional 14 h. Absorbance was measured at 490 nm using a microplate reader (Varioskan LUX, Thermo Fisher Scientific, Inc., San Jose, CA, USA).

### 4.9. Analysis of Cell Surface Antigens

To differentiate the dHL-60 cells, they were treated with 10 μM ATRA. The cultures were washed, reseeded in fresh media, and then treated with ATRA every third day for up to seven days. After the treatment, the cells were centrifuged, collected, washed, and incubated with an FITC-labeled antibody against CD11b (a surface antigen of dHL-60 cells) for 30 min at 37 °C in the dark. Following centrifugation, the cells were washed twice with PBS and analyzed immediately by flow cytometry using a Laser scanning confocal microscope (LSM880,Carl Zeiss, Inc., Oberkochen, Germany). The proportion of CD11b-positive cells increased in the dHL-60 cells compared with the NC group (Supplementary Figure S3A).

### 4.10. Wright–Giemsa Staining

The HL-60 cells were treated with 10 μM ATRA for 7 days. Then, dHL-60 cell smears were prepared and examined by Wright–Giemsa staining. The stained cells were assessed for size, regularity of the cell margin, and morphological characteristics of the nuclei. The Giemsa staining showed that the HL-60 cells were predominantly myelocytes with round and regular cell margins and large nuclei. A decrease in cell size, denser chromatin, and an increase in the cytoplasm-to-nucleus ratio were observed in the ATRA-treated HL-60 cells. These results indicate that HL-60 cells can be successfully induced to differentiate into dHL-60 cells using ATRA (Supplementary Figure S3B).

### 4.11. Field-Emission Scanning Electron Microscopy (FESEM)

To observe the membrane morphology of the dHL-60 cells, FESEM (Hitachi SU8010, Hitachi Ltd., Tokyo, Japan) images were taken. After treatment of the PMA-induced dHL-60 cells with PF (10, 20, 40 μM) for 4 h, the cells were harvested and used for sample preparation for FESEM. The cell samples were gently washed with sterile PBS, and the biofilms were fixed using a 2.5% glutaraldehyde fixative containing 0.15% alcian blue for 22 h. After fixation, the cells were rinsed again with PBS and dehydrated for 10 min with a gradient of ethanol solutions at different concentrations (30, 50, 70, 80, 90, 95, and 100%). The dehydrated cell samples were dried under a stream of nitrogen, coated with a 5 nm thick layer of platinum, and stored in a vacuum desiccator until they were imaged. The biofilm was observed at an emission voltage of 2 kV.

### 4.12. Immunofluorescence Staining (IF)

Well-conditioned dHL-60 cells were harvested and centrifuged, and the cells were washed three times with PBS. Subsequently, the cells were fixed with 4% paraformaldehyde for 20 min at room temperature. Next, 100 μL of the cell suspension was added to slides pretreated with polylysine. The following routine immunofluorescence staining steps were performed: blocking, incubation with primary antibodies (anti-MPO (dilution: 1:100) and anti-CitH3 (dilution: 1:500)) overnight at 4 °C, incubation with Alexa Fluor TM488 goat anti-mouse IgG antibody (dilution: 1:300) and Alexa Fluor^®^594 goat anti-rat secondary antibody (dilution: 1:1000) for 2 h, and staining with 4′,6-diamidino-2-phenylindole (DAPI) for 10 min. All the samples were imaged using an Olympus FV3000 confocal laser scanning microscope (Axio Zeiss Scope A1, Carl Zeiss, Inc., Oberkochen, Germany).

### 4.13. HepG2 and dHL-60 Cells Co-Culture Experiments

Prior to the co-culture experiments, the HepG2 cells were cultured in RPMI 1640 medium supplemented with 10% FBS until they reached a steady state. Logarithmically grown HepG2 cells were inoculated into the lower chamber, followed by the addition of previously differentiated dHL-60 cells to the upper chamber for incubation. In the first phase, APAP was administered to the HepG2 cells for 6 h following a 24 h pretreatment with PF; subsequently, PMA was administered to the dHL-60 cells for 4 h after a similar pretreatment with PF. The co-culture method was referenced from the previous research work [64,65,66,67].

### 4.14. qRT-PCR

Total RNA was extracted from liver tissues and cells using TRIzol™ Reagent (Thermo Fisher Scientific, Inc., San Jose, CA, USA) in accordance with the manufacturer’s instructions. The cDNA synthesis of mRNA was conducted utilizing the AG Reverse Transcription Reagent Premix Solution (Accurate Biotechnology (Hunan) Co., Ltd., Chasngsha, China) following the manufacturer’s instructions after determining the mRNA concentration. A qRT-PCR analysis was conducted in accordance with the manufacturer’s instructions. β-actin served as the normalization control for the mRNA expression levels of the target genes. The PCR primer sequences are provided in Table S2 in the Supplementary Materials.

### 4.15. WB

Liver tissues were lysed in radioimmunoprecipitation assay buffer for protein extraction. Following quantification using a Bicinchoninic Acid protein assay kit, the protein samples were separated using 10–15% SDS-PAGE gel electrophoresis and subsequently transferred onto a polyvinylidene difluoride membrane. After the bands were blocked for one hour in 5% skim milk at 37 °C, the bands were incubated with different primary antibodies, such as antibodies against NLRP3, caspase-1, GSDMD, MPO, PADI4, NE, and CitH3, at 4 °C overnight. The membrane was washed three times with TBST for 5 min each and then incubated with a secondary antibody (1:3000 dilution) for two hours at 37 °C. Finally, the membrane was developed using an enhanced chemiluminescence reagent. The protein levels on the membrane were analyzed using β-actin as an internal reference control. A list of the antibodies utilized in this study is presented in Table S3 in the Supplementary Materials.

### 4.16. Statistical Analysis

The data are shown as the mean ± standard deviation (SD), and the statistical significance was established at *p* < 0.05. All data were analyzed using GraphPad Prism 9. The normality of the distribution was analyzed using the Kolmogorov–Smirnov test. Since the data passed the tests for normality and homogeneity of variance, parametric tests were used. For comparisons between the two groups, an independent samples t-test was used to assess the difference. A *p*-value of < 0.05 was considered statistically significant. A one-way analysis of variance (ANOVA) was performed to assess the difference among the mean values of more than two independent groups, and the significant differences between the groups are indicated with different letters.

## 5. Conclusions

In summary, PF showed significant effects on AILI, both in vivo and in vitro, by intervening in the crosstalk between hepatocyte pyroptosis and NETs. Additionally, we found that HMGB1 plays a critical role in mediating the crosstalk between hepatocyte pyroptosis and NETs. Thus, PF has the potential to be developed as a topical drug for treating AILI. It is worth noting that the current research still has several limitations, and the molecular mechanism through which PF regulates the crosstalk between hepatocyte pyroptosis and NETs still needs to be explored further.

## Data Availability

The data that support the findings of this study are available from the corresponding authors upon reasonable request.

## Supplementary Materials

The supporting information can be downloaded at: https://www.mdpi.com/article/10.3390/ijms26041493/s1.

## Abbreviations

ALF	Acute liver failure
ALT	Alanine transaminase
AML-12	Alpha mouse liver 12 cells
APAP	Acetaminophen
AST	Aspartate transaminase
ATRA	All-trans retinoicacid
caspase-1	Cysteine-aspartic protease 1
CitH3	Citrullinated histone H3
dHL-60	Neutrophil-like differentiated HL-60 cells
DNase I	Deoxyribonuclease I
DSF	Disulfiram
ELISA	Enzyme-linked immunosorbent assay
FBS	Fetal bovine serum
FESEM	Field-emission scanning electron microscopy
GSDMD	Gasdermin D
HepG2	Human hepatoma G2 cells
HL-60	Human promyelocytic leukemia cells
HMGB1	High-mobility group box 1
H&E	Hematoxylin and eosin
IL-18	Interleukin-18
IL-1β	Interleukin-1β
IL-6	Interleukin-6
LDH	Lactate dehydrogenase
MDA	Malondialdehyde
MPO	Myeloperoxidase
MTT	Thiazolyl blue tetrazolium bromide
NAC	N-acetylcysteine
NE	Neutrophil elastase
NETs	Neutrophil extracellular traps
NLRP3	NOD-, LRR-, and pyrin domain-containing protein 3
PF	Paeoniflorin
PADI4	Peptidyl arginine deiminase 4
PBS	Phosphate buffer saline
PMA	Phorbol 12-myristate 13-acetate
qRT-PCR	Quantitative real-time polymerase chain reaction
TNF-α	Tumor necrosis factor alpha
