# Supplementary material for: Paeoniflorin Attenuates APAP-Induced Liver Injury via Intervening the Crosstalk Between Hepatocyte Pyroptosis and NETs
Source: Int J Mol Sci. 2025 Feb 11;26(4):1493. doi: 10.3390/ijms26041493 (PMC11855121; doi:10.3390/ijms26041493)
Supplement: Supplementary file 1 [file ijms-26-01493-s001.zip › ijms-3430012-supplementary.pdf]

Supplementary Material

Paeoniflorin Attenuates APAP-Induced Liver Injury via Intervening the Crosstalk Between Hepatocyte Pyroptosis and NETs

Yu-Ru Zhu [1,†] Ya-Qin Yang [1,†], Dan-Dan Ruan [1], Yue-Mei Que [1], Hang Gao [2], Yan-Zi Yang [1,*] and Hua-Jun Zhao [1,2,*]

1 School of Pharmaceutical Sciences, Zhejiang Chinese Medical University, Hangzhou, Zhejiang 311402, China; zyr@zcmu.edu.cn (Y.-R.Z.); rc.yaqin0902@zcmu.edu.cn (Y.-Q.Y.); 202321126811210@zcmu.edu.cn (D.-D.R.); 202321124011142@zcmu.edu.cn (Y.-M.Q.)

2 Academy of Chinese Medical Sciences, Zhejiang Chinese Medical University, Hangzhou 310053, China

* Correspondence: yangyanzi@zcmu.edu.cn (Y.-Z.Y.); zhj@zcmu.edu.cn (H.-J.Z.)

† These authors contributed equally to this work.

Table of Contents

Supplementary tables

Table S1. Materials.

Table S2. Synthesized primers for qRT-PCR.

Table S3. Antibodies.

Supplementary figures

Supplementary Figure S1. APAP inhibits the activity of HepG2 cells.

Supplementary Figure S2. PF attenuates APAP-induced inflammation and pyroptosis in AML-12 cells.

Supplementary Figure S3. ATRA induces differentiation of HL-60 cells into dHL-60 cells.

Table S1: Materials

Name	Catalog No.	Manufacturer
Acetaminophen (APAP)	HY-66005	MCE
N-Acetyl-L-cysteine (NAC)	A9165	Merck
Deoxyribonuclease I (Dnase I)	10104159001	Roche
Disulfiram (DSF)	HY-B0240	MCE
Bicinchoninic acid (BCA) protein determination kits	P0011	Beyotime Biotech In
Alanine aminotransferase (ALT) Assay Kit	C009-2-1	Nanjing Jiancheng Biotechnology Research Institute Co., Ltd.
Aspartate aminotransferase (AST) Assay Kit	C010-2-1	Nanjing Jiancheng Biotechnology Research Institute Co., Ltd.
Lactate dehydrogenase (LDH) assay kit	A020-2-2	Nanjing Jiancheng Biotechnology Research Institute Co., Ltd.
Malondialdehyde (MDA)assay kit	A003-1-2	Nanjing Jiancheng Biotechnology Research Institute Co., Ltd.
Myeloperoxidase (MPO) assay kit	A044-1-1	Nanjing Jiancheng Biotechnology Research Institute Co., Ltd.
Mouse High-mobility group box 1 (HMGB-1) ELISA Kit	E-EL-M0676	Elabscience Biotechnology Co., Ltd.
Human HMGB-1 ELISA Kit	E-EL-H1554	Elabscience Biotechnology Co., Ltd.
Mouse Neutrophil extracellular trap network (NETs) ELISA Kit	MM-1183M1	Jiangsu Meimian Industrial Co., Ltd.
Human Neutrophil extracellular trap network (NETs) ELISA Kit	ml060524	Enzyme linked biology Co., Ltd.
Hematoxylin and Eosin (H&E) Staining Kit	C0105	Beyotime Biotech Inc
Goat Anti-Mouse IgG	ab150113	Abcam
Goat Anti-Rabbit IgG	ab150080	Abcam
APC anti-mouse/human CD11b	101211	BioLegend, Inc.
Wright-Giemsa Stain Solution	G1020	Solarba Technology Co., Ltd.
Stroke-physiological Saline Solution (SPSS)	ST341	Beyotime Biotech Inc
4% Paraformaldehyde Fix Solution	P0099	Beyotime Biotech Inc
FBS	10099141	Thermo Fisher Scientific
Dulbecco's Modified Eagle Medium (DMDE)	11995065	Thermo Fisher Scientific
Roswell Park Memorial Institute (RPMI) 1640	11875-093	Thermo Fisher Scientific
All-trans retinoicacid (ATRA)	R2625	Merck
Phorbol 12-myristate 13-acetate (PMA)	P8139	Merck
Methylthiazolyldiphenyl (MTT)	475989	Merck

TRIzol™	15596026CN	Thermo Fisher Scientific
Evo M-MLV RT Premix for qPCR	AG11706	Accurate Biotechnology (Hunan) Co., Ltd.

Table S2: Synthesized primers for qRT-PCR

Gene	Species	Forward	Reverse
IL-1β	human	5′-CCACCTCCAGGGACAGGATA-3′	5′-TGGGATCTACACTCTCCAGC-3′
IL-1β	mouse	5′-GAAATGCCACCTTTTGACAGTG-3′	5′-TGGATGCTCTCATCAGGACAG-3′
TNF-α	human	5′-CCTCTCTCTAATCAGCCCTCTG-3′	5′-GAGGACCTGGGAGTAGATGAG-3′
TNF-α	mouse	5′-CAGGCGGTGCCTATGTCTC-3′	5′-CGATCACCCCGAAGTTCAGTAG-3′
IL-6	human	5′-CCTGAACCTTCCAAAGATGGC-3′	5′-AGTGGTATAGACAGGTCTGTTG-3′
IL-6	mouse	5′-CTGAGGAACCCAGAACTACATCT-3′	5′-GGTAGGCGTCCTTGCCAAT-3′
IL-18	human	5′-TGGCTGCTGAACCAGTAGAAG -3′	5′-TGGCTGCTGAACCAGTAGAAG-3′
IL-18	mouse	5′-CAACTTTGGCCGACTTCACTG-3′	5′-TGGGGTTCACTGGCACTTT-3′
β-actin	human	5′- CCTGGCACCCAGCACAAT -3′	5′- GGGCCGGACTCGTCATAC -3′
β-actin	mouse	5′-AGTGTGACGTTGACATCCGT-3′	5′-TGCTAGGAGCCAGAGCAGTA-3′
NLRP3	human	5′-CGTGAGTCCCATTAAGATGGAG -3′	5′-CGACAGTGGATATAGAACAGA -3′
NLRP3	mouse	5′-ATTACCCGCCCGAGAAAGG-3′	5′-CATGAGTGTGGCTAGATCCAAG-3′

Caspase-1	human	5′-GGCATCTGCGCTCTACCATC-3′	5′-TTTCCGCAAGGTTCGATTTTCA-3′
Caspase-1	mouse	5′-ACAAGGCACGGGACCTATG-3′	5′-TCCCAGTCAGTCCTGGAAATG-3′
GSDMD	human	5′-GTGTGTCAACCTGTCTATCAAGG-3′	5′-CATGGCATCGTAGAAGTGGAAG-3′
GSDMD	mouse	5′-TTCAGGCCCTACTGCCTTCT-3′	5′-GTTGACACATGAATAACGGGGTT-3′
HMGB1	human	5′-TATGGCAAAAGCGGACAAGG-3′	5′-CTTCGCAACATCACCAATGGA-3′
HMGB1	mouse	5′-GGCGAGCATCCTGGCTTATC-3;	5′-GGCTGCTTGTCATCTGCTG-3′

Table S3: Antibodies

Name	Catalog No.	Manufacturer	Dilution for WB
NLRP3 (D4D8T) Rabbit mAb	15101S	Cell Signaling	1/1000
Caspase-1 (E9R2D) Rabbit mAb	83383S	Cell Signaling	1/1000
Gasdermin D (E9S1X) Rabbit mAb	39754S	Cell Signaling	1/1000
HMGB1 Recombinant Rabbit Monoclonal Antibody	ET1601-2	HUABIO	1/1000
β-Actin Antibody	4967S	Cell Signaling	1/2000
Anti-Myeloperoxidase (MPO)	ab208670	Abcam	1/1000
Anti-PADI4 / PAD4	ab96758	Abcam	1/1000
Anti-Neutrophil Elastase (NE)	ab131260	Abcam	1/1000
Citrullinated Histone H3 (Arg17) (E4O3F) Rabbit mAb (Cith3)	97272S	Cell Signaling	1/1000

Supplementary Figure S1. APAP inhibits the activity of HepG2 cells.

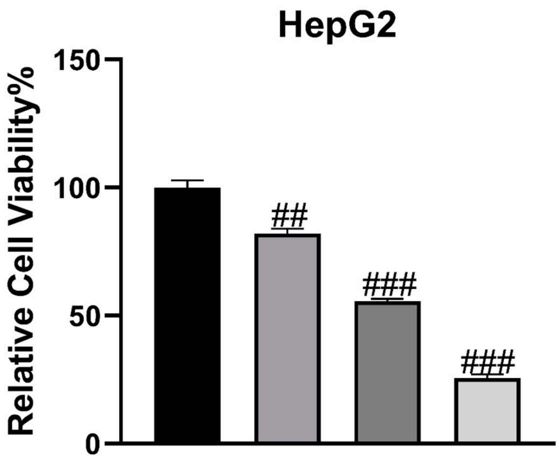

Supplementary Figure S1. APAP inhibits the cell viability of HepG2 cells. (A) HepG2 cells were treated with APAP (10 mM) for 0, 6, 12, and 24 hours, after which the cell viability was determined using MTT (n=3). All experimental data were presented as mean ± SD. ## $p < 0.01$, ### $p <0.001$ vs. control group.

Supplementary Figure S2. PF attenuates APAP-induced inflammation and pyroptosis in AML-12 cells.

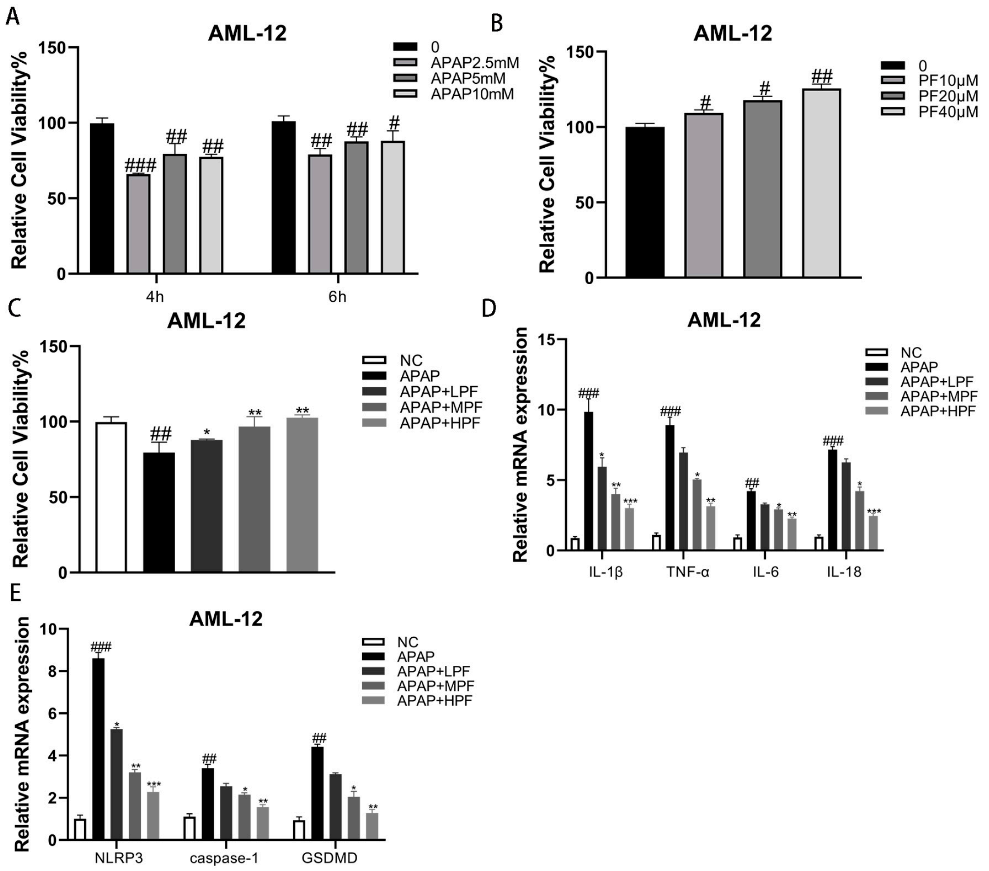

Supplementary Figure S2. PF attenuates APAP-induced inflammation and pyroptosis in AML-12 cells. (**A**) AML-12 cells were treated with various concentrations of APAP (0, 2.5, 5, 10 mM) for 4 and 6 hours respectively, after which the cell viability was determined using MTT (n=3); (**B**) AML-12 cells were treated with various concentrations of PF (0, 10, 20, 40 μM) for 24 hours; subsequently, cell viability was assessed using the MTT assay (n=3); (**C**)AML-12 cells were pretreated with various concentrations of PF (0, 10, 20, 40 μM) for 24 hours, and then treated with APAP (5 mM) for 4 hours, after which the cell viability was assessed using MTT (n=3); (**D**) The mRNA expression levels of IL-1β, TNF-α, IL-6 and IL-18 in AML-12 cells were quantified using qRT-PCR; (**E**) The mRNA expression levels of NLRP3, caspase-1, GSDMD and HMGB1 in AML-12 cells were quantified using qRT-PCR. All experimental data were presented as mean ± SD. ## $p < 0.05$, ## $p < 0.01$, ### $p < 0.001$ vs. control group; * $p < 0.05$, ** $p <$

0.01 and *** $p < 0.001$ vs. APAP group.

Supplementary Figure S3. ATRA induces differentiation of HL-60 cells into dHL-60 cells.

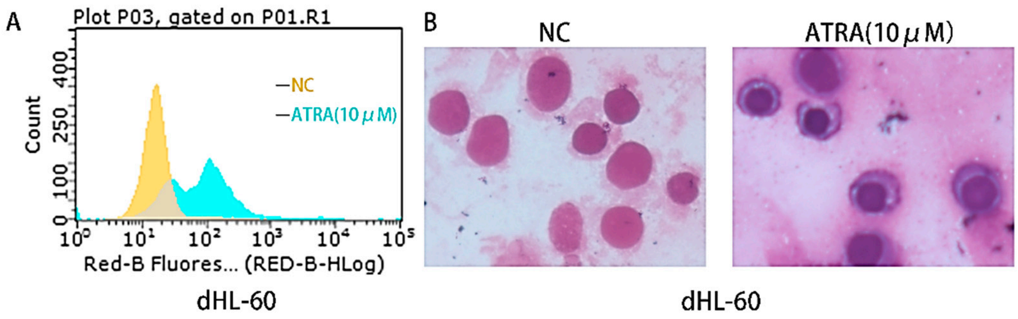

Supplementary Figure S3. ATRA induces differentiation of HL-60 cells into dHL-60 cells.

(**A**) The expression levels of CD11b in dHL-60 cells were detected by flow cytometry; (**B**) The dHL-60 cells morphology was assessed with Giemsa staining and analyzed under a light microscope (magnification: × 400).
